# Conserved methionine 165 of matrix protein contributes to the nuclear import and is essential for influenza A virus replication

**DOI:** 10.1186/s12985-018-1056-x

**Published:** 2018-12-03

**Authors:** Petra Švančarová, Tatiana Betáková

**Affiliations:** Biomedical Research Center - Slovaks Academy of Sciences, Institute of Virology, Bratislava, Slovak Republic

**Keywords:** Influenza a virus, M1 protein, NP protein, CLUH, Reverse genetic

## Abstract

**Background:**

The influenza matrix protein (M1) layer under the viral membrane plays multiple roles in virus assembly and infection. N-domain and C-domain are connected by a loop region, which consists of conserved RQMV motif.

**Methods:**

The function of the highly conserve RQMV motif in the influenza virus life cycle was investigated by site-directed mutagenesis and by rescuing mutant viruses by reverse genetics. Co-localization of M1 with nucleoprotein (NP), clustered mitochondria homolog protein (CLUH), chromosome region maintenance 1 protein (CRM1), or plasma membrane were studied by confocal microscopy.

**Results:**

Mutant viruses containing an alanine substitution of R163, Q164 and V166 result in the production of the virus indistinguishable from the wild type phenotype. Single M165A substitution was lethal for rescuing infection virus and had a striking effect on the distribution of M1 and NP proteins. We have observed statistically significant reduction in distribution of both M165A (p‹0,05) and NP (p‹0,001) proteins to the nucleus in the cells transfected with the reverse –genetic system with mutated M1. M165A protein was co-localized with CLUH protein in the cytoplasm and around the nucleus but transport of M165-CLUH complex through the nuclear membrane was restricted.

**Conclusions:**

Our finding suggest that methionine 165 is essential for virus replication and RQMV motif is involved in the nuclear import of viral proteins.

## Background

Influenza A virus is a major respiratory pathogen. The genome contains eight segments of negative sense, single stranded RNA, each encapsidated into ribonucleoproteins by the viral RNA-dependent RNA polymerase and multiple copies of the viral nucleoprotein. The approximately 13 kb genome encodes up to 16 proteins [1–4].

The matrix protein M1 is a multifunctional protein playing many essential roles throughout the virus life cycle. M1 protein is a major structural protein, which associates with ribonucleoprotein and the cytoplasmic tails of haemagglutinin and neuraminidase by forming a matrix layer underneath the lipid bilayer of the viral envelope [5, 6]. This protein plays key roles in structural integrity, replication, virus assembly and budding [7, 8].

The M1 protein is encoded by the M gene segment and comprised of 252 amino acids. This α-helical protein consists of two domains: N-terminal domain from 1 to 165 aa and C-terminal domain from 165 to 252 aa, which are linked by a protease sensitive loop. Although the crystallisation attempts on the full-length of M1 protein, only the structures of N-domain could be determined as the C-terminal region was digested during crystallisation and therefore missed the final crystal [5, 9]. The N-terminal fragment has a globular structure and consists of nine α-helices and eight loop regions, and does not include any β-strands. According to analysis using programs for secondary structure prediction, the C-domain consists of three α-helical regions. N-domain and C-domain are connected by a loop region, which forms small α-helix (aa 162–166). The conservative MQMV motif presented in the α-helix possesses a glutamine-methionine cleavage site [10].

The multiple functional motifs (aa 91–105) includes nuclear localization signal (NLS) (RKLKR) involved in translocation of M1 from the cytoplasm into the nucleus and in binding of vRNA and vRNP [11, 12]. The zinc finger motif (aa 148–162) has been shown to associate with zinc molecules to inhibit viral replication, and is important for interaction with RNP [13–15]. The arginine triplet R76/77/78 participate in M1 interaction with membranes [16]. The oligomerization pattern of M1 is controlled by residues 181–193 and forms highly conserved region in all subtypes of influenza A virus [17]. N-terminal domain of M1 mediates protein-protein contact with NP [11, 18]. Baudin et al. [19] found that C-terminal domain bounds to vRNA and NP alone whilst Noton et al. [20] concluded that for the binding of NP is primarily responsible the middle domain of M1. Interaction of M1 with vRNPs is crucial for nuclear export of vRNPs-M1-NEP complex via CRM1-dependent pathway [7, 19, 21–25].

In the present study, we have investigated the role of the putative glutamine-methionine cleavage site in the RQMV motif in replication. The RQMV motif is conserved in all influenza A strains. We have used the site-direct mutagenesis and rescue of mutant viruses by reverse genetic systems to define the function of this region in the virus life cycle. Crucially, we showed that alanine substitution of methionine 165 was lethal for virus replication and affected the distribution of viral and cellular proteins.

## Materials and methods

### Cells and viruses

293 T (ATCC CRL-3216) and MDCK (ATCC CCL-34) cells were grown in Eagle’s Minimum Essential Medium (EMEM) containing 10% calf serum in a 5% CO_2_ atmosphere at 37 °C. The rescued viruses were growth on MDCK cells. Viral titers were determined by plaque assay.

### Antibodies

Mouse monoclonal antibody raised against anti-influenza A, Puerto Rico 8/34 (H1N1), Bangkok 1/79 (H3N2), matrix protein (I7650-19C) was purchased from US Biological. The rabbit polyclonal anti-CRM1 antibodies (ab24189), anti-NP antibodies (ab104870), and anti-sodium potassium ATPase antibody(ab197713) – plasma membrane marker (AlexaFluor488) were obtained from Abcam, rabbit polyclonal anti-CLU1/ KIAA0664 antibody (LS-C288546) was ordered from LS BIO. Rabbit antisera were produced to the peptide corresponding to the C-terminal sequence SAVDVDDGHFVNIELE of the M2 protein conjugated to keyhole limpet haemocyanin [26]. Alexa Fluor 555 goat anti-mouse IgG (H + L) (A11008) and Alexa Fluor 488 donkey anti-rabbit IgG (H + L) (A31572) (Invitrogen) were used as secondary antibodies for immunofluorescence microscopy.

### Plasmid construction

Viruses with mutated M1 protein were generated using the plasmid-based reverse genetics system, a kindly provided by Dr. Y. Kawaoka [27]. The eight plasmids contain the cDNA of the virus A/WSN/33 (H1N1). Plasmids pHW187-M encoding the mutant M1 proteins R163A, Q164A, M165A, and V166A were prepared using Phusion Site-Directed Mutagenesis Kit (Finnzymes). Sequences of oligonucleotide primers and details of cloning are available upon request. All constructs were sequenced to confirm that they lacked unwanted mutations. We also confirmed the functionality of the resulting plasmids by reverse genetics. DNA was purified using Pure Yield™ Plasmid Maxiprep (Promega).

### Generation of viruses by reverse genetics

Plasmid-based reverse genetics for virus generation were performed as previously described [25]. Briefly, the eight plasmid containing the cDNA of the virus polI –polII system for generation of A/WSN/33 (H1N1) virus were transfected into co-cultured 293 T and MDCK cells using TurboFect Transfection reagent (Thermo Scientific). Plasmids pHW187-M encoding the mutant M1 proteins R163A, Q164A, M165A, and V166A were used to rescue of mutant viruses. At 48 h posttransfection, culture supernatants were harvested and inoculated into MDCK cells for virus propagation. After 48 h, cell culture media were centrifuged to remove cell debris, and the supernatants were stored as stock viruses. The titers of the stock viruses were determined by plaque assays in MDCK cells as was previously published [28]. All viruses were sequenced to confirm the presence of wanted mutations. Prepared A/WSN/33 virus was used as control wild type virus (VC).

### Western blotting

The cell sediment was lysed in extraction buffer (1% Triton-X-100, 1mMEDTA, 20mMTris-HCl, pH 7.4) containing proteinase inhibitor Sigma Fast Protease Inhibitors (Sigma). After 10 min on ice, the lysates were clarified by microcentrifugation for 1 min, and the supernatants were analysed by electrophoresis on 12.5% polyacrylamide gels. Immunoblotting was done as described by Betakova et al. [29] using rabbit anti-M2, Protein A-horseradish peroxidase conjugate (BioRad) and 3,3′-Diaminobenzidine (DAB) Enhanced Liquid Substrate System tetrahydrochloride (Sigma).

### Immunofluorescence assay

Co-cultured 293 T and MDCK cells were grown on glass coverslips and transfected with eight RNA polymerase I plasmids as described above. After 24 h, the cells were fixed with 3% paraformaldehyde or methanol, permeabilised with 0.01% Triton in phosphate-buffered saline (PBS) and immunolabelled with primary antibodies (1 μg/ml) diluted in PBS containing 1% bovine serum albumin (BSA). Primary antibodies were visualised using fluorescein or rhodamine conjugated secondary antibodies diluted in PBS, 1% BSA. The nuclei were labelled for 10 min with DAPI.

### Microscope image acquisition

Confocal laser scanning was performed using a Zeiss LSM 510 Meta fitted with a Plan Apochromatic × 40/1.4 oil objective lens. The Z-stack images were taken in a range from 0.1 to 0.5 μm. Images were collected at 16 bits and at a resolution of 1024 by 1024 pixels. The image processing and analysis were carried out using Fiji/ImageJ software.

### Statistical analyses

Significant differences in the expression of proteins between the control group (M1) and M165A were calculated using the unpaired Student‘s t-test. *P* values < 0.05 were considered to be significant. Statistical analysis was performed using Graph-Pad Prism software.

## Results

### M165 is essential for virus replication

**Fig. 1:**
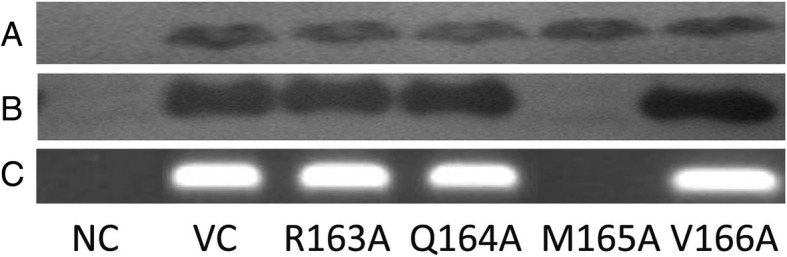
Confirmation of effective transfection in co-cultured 293 T and MDCK cells and virus production in MDCK cells. The expression of viral proteins was detected in transfected co-cultured 293 T and MDCK cells (**a**) and in re-infected MDCK cells (**b**) at 48 h post transfection/infection by western blotting with anti-M2 polyclonal antibodies. (**c**) Detection of mRNA M1 in infected MDCK cells. The MDCK cells were infected with supernatant from co-cultured 293 T and MDCK cells. At 48 h post-infection, the cell lysates were used for RNA purification and mRNA specific to M1 was determined by RT-PCR

To test the role of single amino acids in the RQMV motif, we have generated plasmids with single mutation in M1 gene. The recombinant viruses were rescued by transfection in co-cultured 293 T and MDCK cells. Since anti-M1 antibody did not work in western blot, we used anti-M2 polyclonal serum to compare expression of M2 protein after transfection with M1 (control virus – VC), R163A, Q164A, M165A, and V166A plasmids (Fig. 1a). Mutation in M1 protein did not affect expression of M2 protein. Although M2 protein could not be used for M1 protein measurement, it was used to compare transfection efficiency. The expression of M1 was checked by indirect fluorescent microscopy (data not shown). The expression of M1 and M2 proteins from all plasmids was comparable. All viruses were rescued except M165A mutant, when the M2 protein was not detected in MDCK cells re-infected with medium from transfected cells (Fig. 1b). The lack of M165A virus replication in re-infected MDCK cells was confirmed by RT-PCR. The viral RNA was detected only in the MDCK cells re-infected with supernatant from VC, R163A, Q164A, and V166A mutants (Fig. 1c).

### Replication of rescued mutant viruses is comparable with control virus

**Fig. 2:**
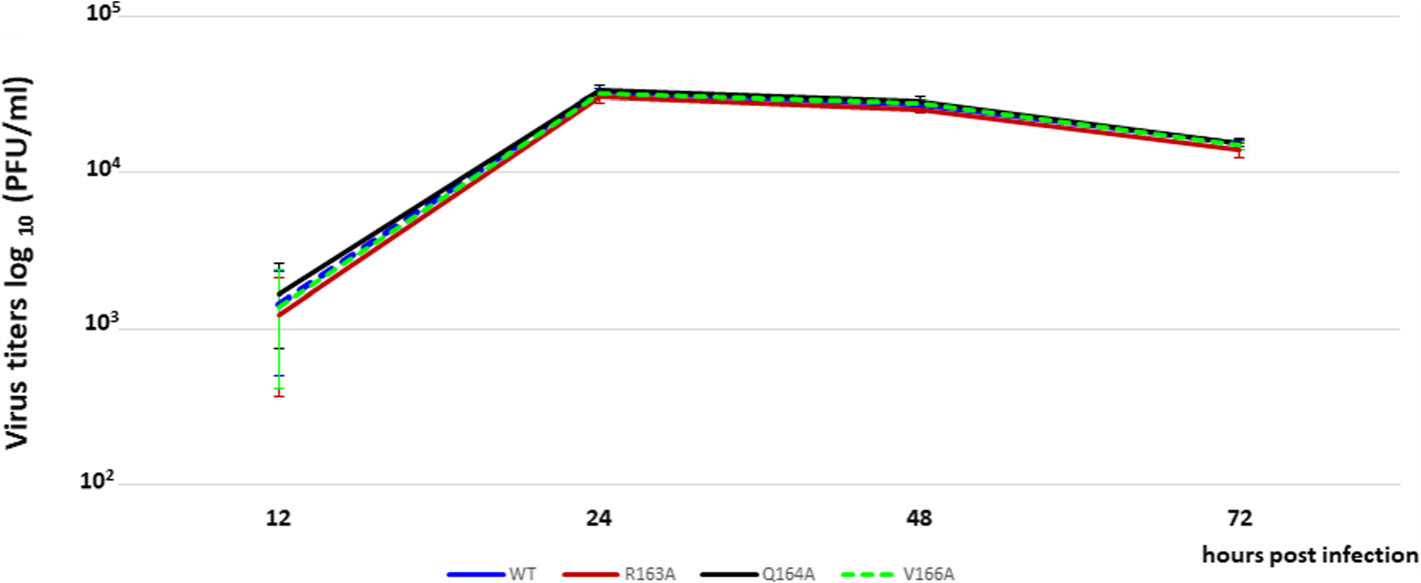
Multistep virus growth curve. The virus production was determined in multistep growth curve (MOI = 0,01) of control virus (VC) and virus-containing R163A, Q164A, and V166A mutation. The culture supernatants collected at the indicated time points were subjected to plaque assays for virus titration. Error bars represent standard deviation of three independent experiments

Generated single point M1 mutants were further characterized. The growth kinetics of these viruses was determined using MDCK cells. There were no obvious differences among control wild type virus (VC) prepared by reverse genetic system and R163A, Q164A and V166A mutant viruses in the growth curves (Fig. 2). The appearance of revertants was not observed after 6 passages in MDCK cells.

### M165A blocked transport of NP into the nucleus

**Fig. 3:**
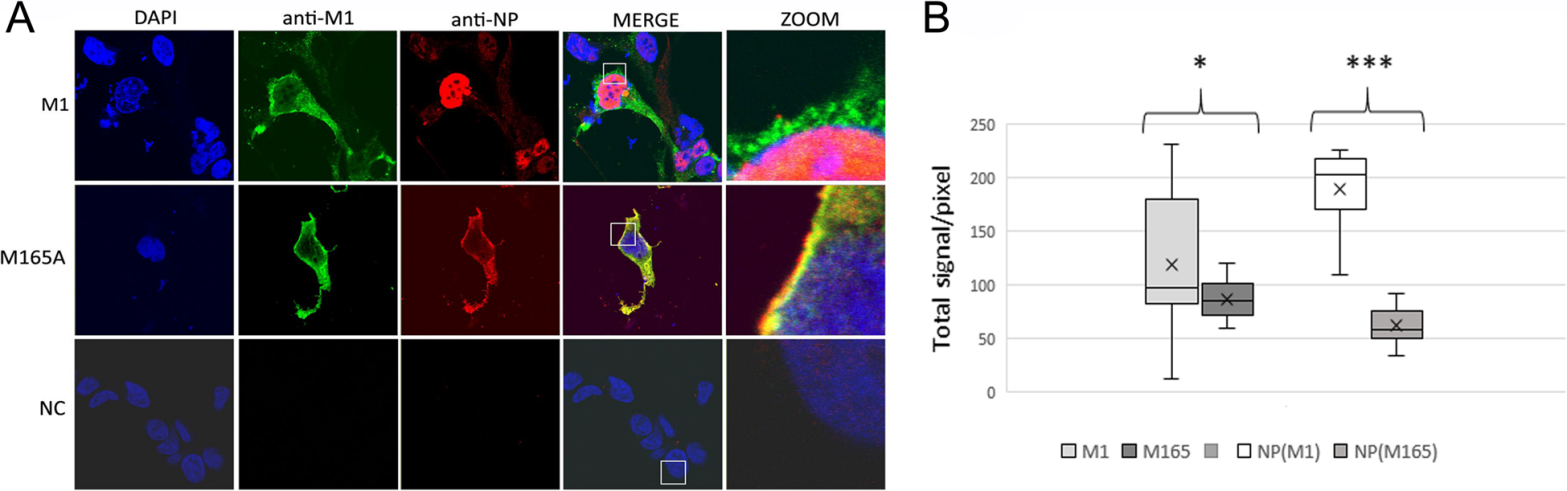
Cellular co-localization and quantification of NP with M1 and M165A mutant using immunofluorescence confocal microscopy. **a/** Co-cultured 293 T and MDCK cells were transfected with eight plasmids for reverse genetic system. After 24 h, the cells were fixed and M1 and M165A were detected using a primary anti-M1 antibody and a secondary antibody coupled to Alexa488 (in green) and NP protein was labelled with anti- NP antibody and a secondary antibody coupled with Alexa555 (in red). Nuclei were stained with DAPI (in blue). NC represents non transfected cells. The intracellular distribution of M1 and NP was imaged by confocal laser scanning fluorescence microscopy (LSM Zeiss 510 Meta). Insets show higher magnification views of the selected areas. The results are representative of three independent experiments. **b/** The images obtained from fluorescent microscopy were quantified using Fiji/ImageJ software. The data were delivered from the measurement of 30 cells. The graph indicated mean ± SD. Statistical analysis was performed using t-test. A *p*-value < 0.05 was considered statistically significant. NP(M1) is NP in the cells transfected with M1, NP(M165) is NP in the cells transfected with M165A mutant

To understand the role of M165 in virus replication, we examined the effect of M165A mutation on the transport of M1 and NP in transfected cells. Eight RNA polymerase I plasmids (reverse genetic) were transfected into co-cultured 293 T and MDCK cells. At 24 h posttransfection, the cells were fixed and labelled with anti-M1 and anti-NP as described in material and methods. M1 and M165A were observed in the cytoplasm and nucleus. There were differences in the proportion of M1 and M165A mutated protein in the nuclei of transfected cells (Fig. 3a). The pictures obtained by confocal fluorescent microscopy were analysed using Fiji/ImageJ software and the total signals/pixel in the nucleus was calculated. Compare to M1, the amount of M165A protein was significantly reduced in the nuclei (*p* < 0.05) (Fig. 3b).

Since M1 mediates the binding to NP, we were interested to determine differences in the binding of these two proteins. Subcellular localization of NP protein was examined in the cells transfected with both M1 and M165A proteins. As expected, the predominant nuclear localization of NP was found in the cells transfected with M1 (Fig. 3a). On the other hand, NP expressed in the cells transfected with M165A was co-localized with M165A protein in the cytoplasm and around the nucleus. The quantification analyses showed that the nuclear signal of NP protein in the cells transfected with M1 is significantly lower (*p* < 0.01) in comparison with NP in the cells transfected with M165A (Fig. 3b). Although the co-localization of NP and both M1 and M165A proteins were not disrupted, the migration pattern of M165 and especially NP in the cells transfected with M165A was completely different. Mutation M165A resulted in blockage of NP and M1 transport into the nucleus.

### M165A influences CLUH translocation to the nucleoplasm

**Fig. 4:**
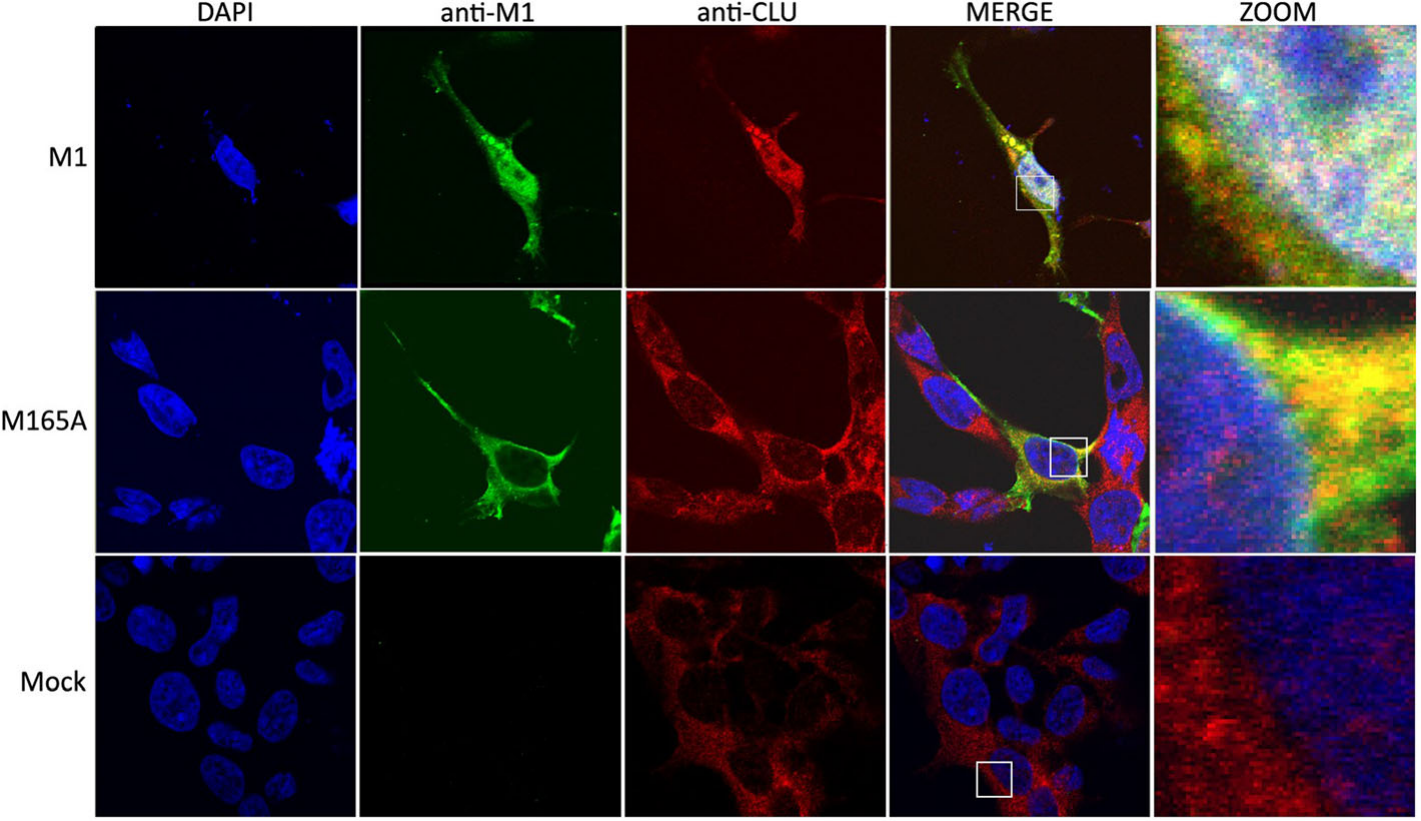
Cellular co-localization of CLUH with M1 and M165A mutant using immunofluorescence confocal microscopy. Co-cultured 293 T and MDCK cells were transfected with eight plasmids for reverse genetic system. After 24 h, the cells were fixed and M1 and M165A were detected using a primary anti-M1 antibody and a secondary antibody coupled to Alexa488 (in green). CLU protein was labelled with anti- CLUH antibody and a secondary antibody coupled with Alexa555 (in red). Nuclei were stained with DAPI (in blue). NC represents non transfected cells. The intracellular distributions of M1 and CLUH were imaged by confocal laser scanning fluorescence microscopy (LSM Zeiss 510 Meta). Insets show higher magnification views of the selected areas. The results are representative of three independent experiments

To determine co-localization M165A and CLUH, co-cultured 293 T and MDCK cells were transfected with eight plasmids for reverse genetic system where M1 was replaced by M165A mutant and CLUH localization was analysed. CLUH plays a role in the subnuclear transport of progeny vRNP to the assembly site of viral nuclear export complexes via nuclear speckles. Normally, CLUH localizes in the cytoplasm. And in influenza virus-infected cells, CLUH translocases from the cytoplasm to the nucleus [30]. In the cells transfected with M1, CLUH was imported into the nucleus (Fig. 4). In M165A transfected cells, transport into the nucleus was impaired and CLUH was co-localized with M165 in the cytoplasm and around the nucleus.

### Association of M165A protein with CRM1 around the nucleus

**Fig. 5:**
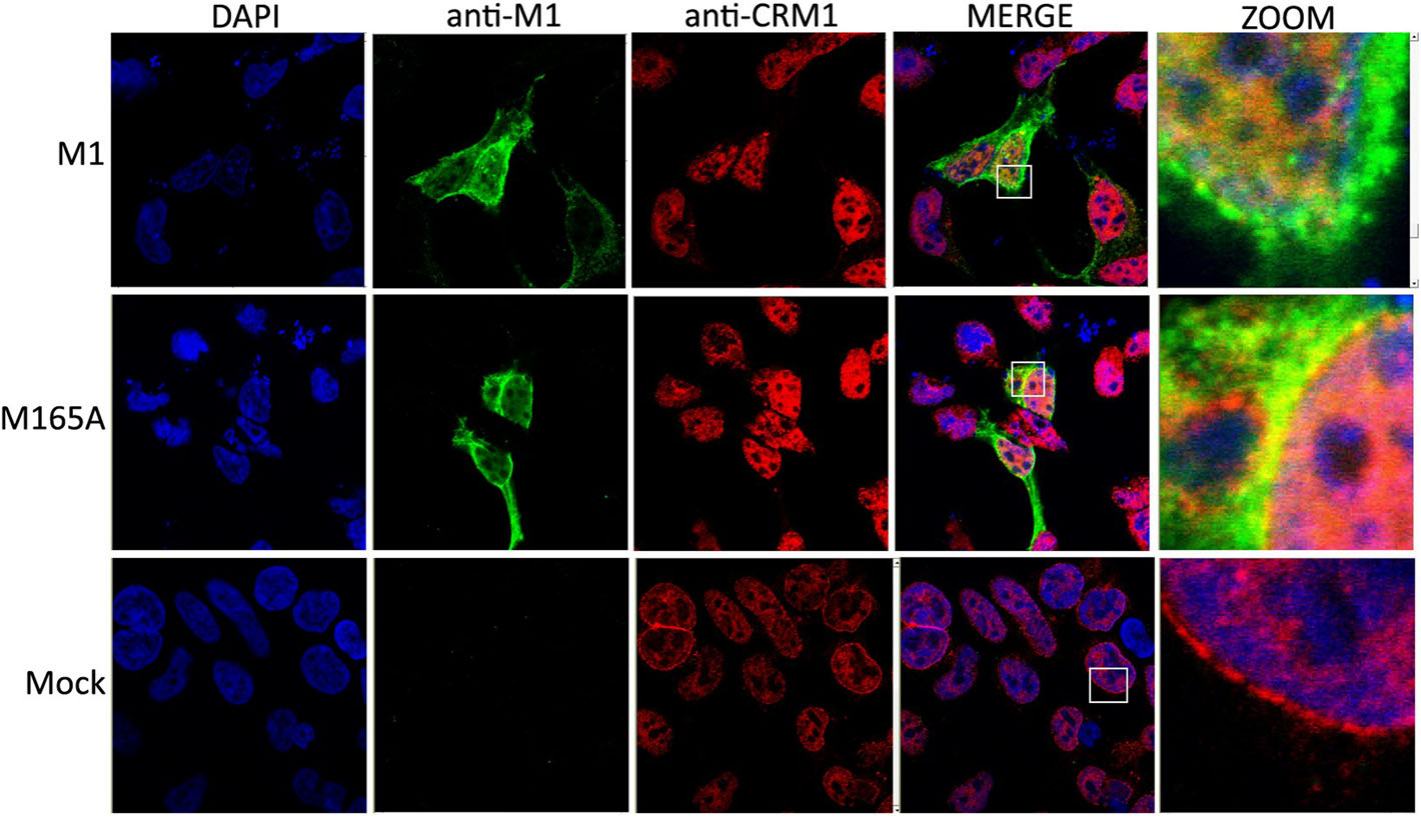
Cellular co-localization of CRM1 with M1 and M165A mutant using immunofluorescence confocal microscopy. Co-cultured 293 T and MDCK cells were transfected with eight plasmids for reverse genetic system. After 24 h, the cells were fixed and M1 and M165A were detected using a primary anti-M1 antibody and a secondary antibody coupled to Alexa488 (in green). CRM1 protein was labelled with anti-CRM1 antibody and a secondary antibody coupled with Alexa555 (in red). Nuclei were stained with DAPI (in blue). NC represents non transfected cells. The intracellular distributions of M1 and CRM1 were imaged by confocal laser scanning fluorescence microscopy (LSM Zeiss 510 Meta). Insets show higher magnification views of the selected areas. The results are representative of three independent experiments

CRM1 has been identified as an export factor for the leucine-rich nuclear export signal (NES) [32]. In untrasfected cells, CRM1 was localized in the nucleus and nuclear membrane was strongly labeled (Fig. 5). Transfection with M1 and M165A resulted in increased amount of the CRM1 protein in the nucleus. Unlike M1 protein co-localized with CRM1 protein in the nucleus, M165A protein was co-localized with CRM1 protein on the cytoplasmic face of the nuclear membrane.

### M165A is associated with the plasma membrane

**Fig. 6:**
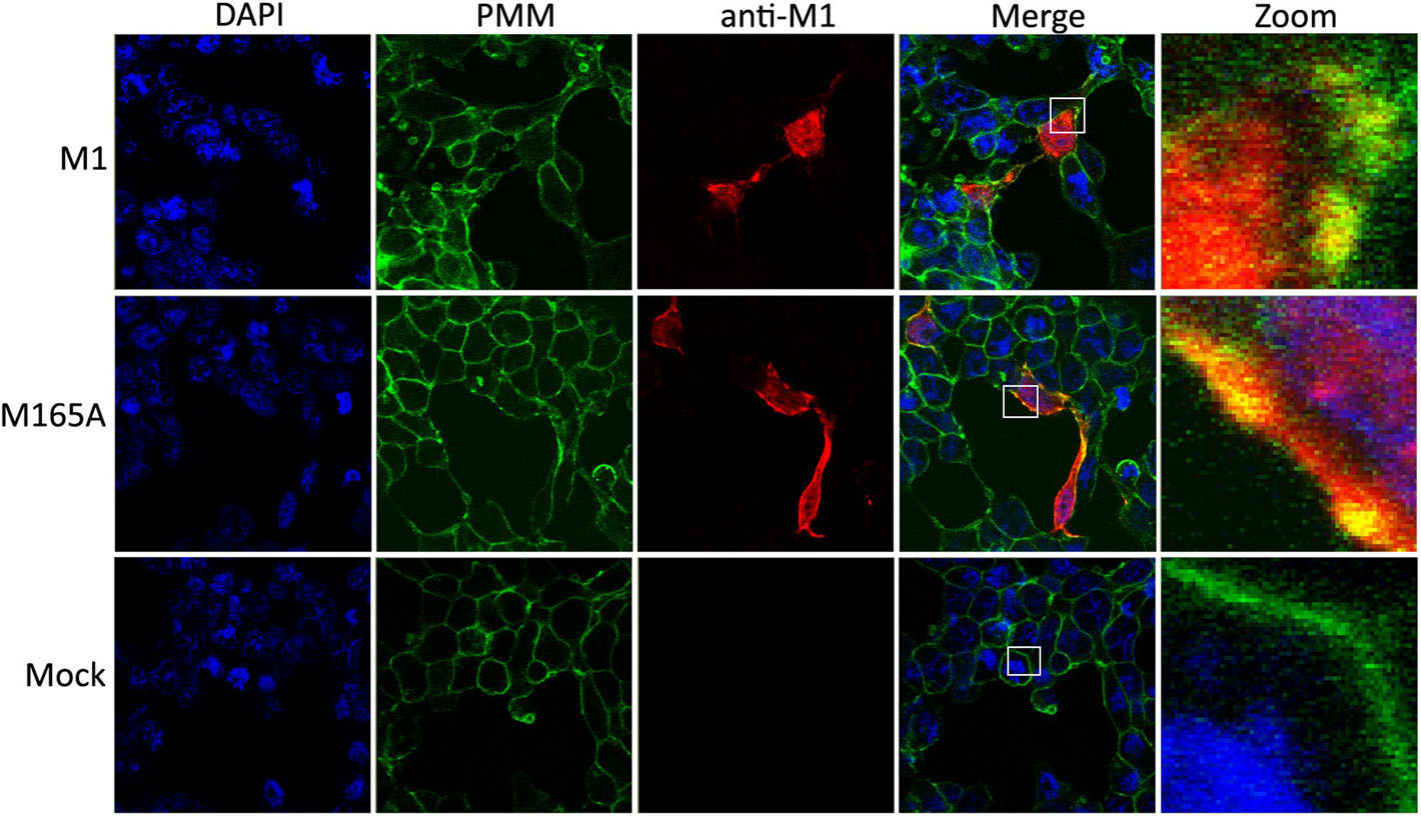
Cellular co-localization of plasma membrane with M1 and M165A mutant using immunofluorescence confocal microscopy. Co-cultured 293 T and MDCK cells were transfected with eight plasmids for reverse genetic system. After 24 h, the cells were fixed with methanol, plasmatic membrane were labelled with anti-sodium potassium ATPase antibody-plasma membrane marker (Alexa Fluor 488) (in green). M1 and M165A were detected using a primary anti-M1 antibody and a secondary antibody coupled to Alexa555 (in red). Nuclei were stained with DAPI (in blue). NC represents non transfected cells, PMM represents plasma membrane. The plasma membrane and intracellular distributions of M1 were imaged by confocal laser scanning fluorescence microscopy (LSM Zeiss 510 Meta). Insets show higher magnification views of the selected areas. The results are representative of three independent experiments

M165A protein was broadly co-localized with plasma membrane. M1 does not normally traffic to the plasma membrane when expressed alone unless it is artificially recruited to the plasma membrane by addition of a targeting peptide or is overexpressed using a vaccinia virus expression system [32, 33]. M1 can be recruited to the plasma membrane by HA or NA via the respective cytoplasmic tail [34, 35], and also by the M2 protein [32]. To determine whether M165A mutation change the M1 presence at the plasma membrane, the co-cultured 293 T and MDCK cells were transfected as described above and plasma membrane were labelled with the anti-sodium potassium ATPase antibody (Fig. 6). Mutation M165A did not interrupt M1 association with the host cellular membrane.

## Discussion

In this study, we have analysed the RQMV motif which is highly conserved among all avian and mammals’ viruses. Alanine substitution for R163, Q164, and V166 in M1 protein did not affect viral growth and distribution of M1 protein inside the cells. Mutation V166A is presented in some avian viruses and thereby it is not surprising that this mutation is well tolerated in our system. Only mutation of M165 to alanine resulted in the failure of virus production. Reverse genetic experiments were repeated more than 20 times and several incubation periods, such as 24, 48, 72 and 96 h were tested. The plasmids were also used in higher concentrations. We have also prolonged the incubation time after re-infection in MDCK cells but M165A mutant virus was never rescued. We have also tried to make a virus with M165 V mutation and this mutation was also lethal.

Mutation M165A in M1 protein had a striking effect on the distribution of NP protein. In the cells transfected with M1, NP protein was localized mainly in the nucleus unlike the NP in the cells transfected with M165A mutant where it was localized mainly in the cytoplasm and around the nuclear membrane. The amount of NP protein found in the nuclei were analysed by one-way ANOVA and t-test. Differences were considered statistically significant at p‹0,001. Surprisingly, differences in localization of M1 protein were less obvious and statistically significant at *p* < 0,05.

Transport of M1 protein to the nucleus is mediated by NLS at amino acids 101–105 [36]. It seems that NLS is not the only one responsible for the M1 transport into the nucleus and other protein-protein interaction are suggested. CLUH, a host protein plays a key role in the subnuclear transport of vRNP. Viral PB2 and M1 induce CLUH translocation to the nucleoplasm and SC35-positive speckles. CLUH is usually cytoplasmic and in virus – infected cells is CLUH translocated from the cytoplasm to the nucleus [30]. In M165A transfected cells, M165A-CLUH complexes were detected mostly in the cytoplasm and around the nucleus. The amount of CLUH detected in the nucleus was much lower than that in M1 transfected cells. The physiological functions of CLUH is not fully understood. Translocation of M1 protein depends on association with the CLUH in cytoplasm. Methionine has the propensity to interact with aromatic-containing residues, including tryptophan, tyrosine, and phenylalanine. The Met-aromatic motif is prevalent in many known protein structures and stabilizes protein-protein binding interactions [37]. M165 may stabilize the structure of M1 protein and might affect its interaction with other cellular proteins.

Interaction of M1 and NP proteins is mainly dependent on the positive-negative charge and is described in connection with the vRNP export from the nucleus [38, 39]. NP is the major protein in the vRNA and plays important role also in vRNP nuclear import [40]. The nuclear transport of NP can be detected in the absence of any other viral proteins in NP transfected cells [41]. Nucleo-cytoplasmic shuttling of NP is mediated through interaction with cellular factors CRM1 and importin-α [42–44]. Interaction of NP with CRM1 is crucial for nuclear export of vRNP-NP-M1-NEP complex [8, 24, 25, 42]. We have found that mutation M165A does not change the distribution and co-localization pattern with importin-α (data not shown). M165A which is not transported into the nucleus and stays near the nucleus where associates with CRM1 present in this region. We suggest that M165A mutation disturbed the transport of CLUH-M165A complex into the nucleus and blocked the CRM1 translocation into the nucleus.

We were not able to study co-localization of other virus proteins with CLUH, CRM-1 or importin-α in the cell transfected with revers genetic plasmids including M165A mutant since we did not possess suitable antibodies for multiple labelling and transfection with eight different plasmids did not ensure expression of all proteins in one cell. However, we have observed the same distribution of M1 and M165A proteins in the cells transiently co-transfected with plasmid encoding only M1 protein with or without plasmid encoding NP protein, as well as in the cells transfected with five plasmids: pHW182-PB1, pHW181-PB2, pHW183-PA, pHW185-NP, and pHW127M. M1 protein is not usually transported to the plasma membrane when expressed alone unless it is recruited to the plasma membrane by HA, NA, and M2 proteins [32, 34, 35]. Since co-localization of M165A protein with the plasma membrane is not impaired, we assume that neither is affected the transport and interaction of M165A with HA, NA, and M2 proteins.

Although M165A protein accumulates around the nucleus where is co-localized with CLUH and CRM1 proteins, the transport into the nucleus is restricted. We suggest that this restriction influence also transport of NP to the nucleus. There is very close relationship between NP and C-domain (residue 165–252) of M1 and mutation in this region might affect the interaction between NP-M and formation of nuclear export complex. Mutation in M1 and NP can cause defects in nuclear export [14, 46]. Transport of NP protein can be restricted by accumulated CLUH-M165A and CRM1-M165A complexes on the nuclear membrane and/or some cellular proteins involved in the transport could be dysregulated. Still, there is many studies focusing on nuclear export but nuclear import is not fully explained yet. It was suggested that some still unknown viral and host nuclear factors must be involved in regulation transport of vRNP and viral proteins, and our findings support these suggestions [31, 45, 46].

## Conclusions

We studied the role of RQMV motif in virus replication. Single R163A, Q164A and V166A substitution resulted in the production of viable viruses. Mutation of M165 was lethal and affected transport of M165A and NP proteins to the nuclei of transfected cells. These data suggest that highly conserved RQMV motif is involved in the nuclear import of viral proteins and other mechanism for the subnuclear transport of viral and host factors must exist. Further analyses should be undertaken to identify the mechanism, and M165 might be a potential target for the development of anti-influenza virus drugs.

## Abbreviations

CLUH: Clustered mitochondria homolog protein
CRM1: Chromosome region maintenance 1
M1: Matrix protein
NES: Nuclear export signal
NLS: Nuclear localisation signal
NP: Nucleoprotein
VC: Control virus A/WSN/33 prepared by reverse genetic
vRNA: (influenza genomic) viral RNA
